# Simulation of gas transport in a landfill with layered new and old municipal solid waste

**DOI:** 10.1038/s41598-021-88858-5

**Published:** 2021-05-03

**Authors:** Tao Zhang, Jianyong Shi, Xun Wu, Hai Lin, Xiulei Li

**Affiliations:** 1 School of Civil Engineering and Architecture, Nanchang Hangkong University, Nanchang, 330063 China; 2 Geotechnical Engineering Research Institute, Hohai University, Nanjing, 210098 China; 3 Key Laboratory of Ministry of Education for Geomechanics and Embankment Engineering, Hohai University, Nanjing, 210098 China; 4 School of Civil Engineering and Architecture, Nanchang University, Nanchang, 330031 China; 5 College of Hohai, Chongqing Jiaotong University, Chongqing, 400074 China

**Keywords:** Environmental sciences, Solid Earth sciences

## Abstract

Average biodegradation rate of newly filled municipal solid waste (MSW) in landfills is relatively fast, and the landfill gas produced by the new MSW biodegradation can cause great variations in gas pressure. To predict the gas pressure distribution in the MSW layer, a one-dimensional gas transport model is established in this study. The following factors are considered in this model: (1) the variation of gas permeability with depth; (2) the anisotropy ratio of gas permeability; (3) the settlement caused by waste biodegradation. Furthermore, a single peak model for gas production is applied as the source term of gas production. The equation for settlement caused by waste biodegradation is presented, and the time of peak gas production rate is obtained by fitting the settlement of the newly filled layer. The stratification of the unsaturated and saturated regions is taken into account by distinguishing the difference in gas saturation. The layering of the new and old waste layers is considered by distinguishing the difference in the length of time that waste has been degraded to produce gas. Based on the method of numerical calculation, the gas pressure distribution in the landfill with layered new and old MSW is well simulated. The position where the maximum gas pressure occurs is found. The sensitivity analysis shows that the influence of the anisotropy ratio on gas pressure distribution is more significant.

## Introduction

The continuous generation of gas in landfills causes landfill gas to escape into the atmosphere due to pressure differences^1–4^. Additionally, slope instability caused by changes in landfill gas pressure often occurs^5–7^. Therefore, the exploration of gas pressure and its distribution in landfills has become an important research area. Kjeldsen and Fischer^8^ monitored the gas pressure in the old waste layer of Skellingsted landfill for 35 days, and results show that the variation of gas pressure in the landfill has a great influence on the composition of landfill gas. Spokas and Bogner^9^ and Bentley et al.^10^ measured gas pressure in the Olinda and Louisiana landfills for 3 and 5 days, respectively, and found that the measured pressure is influenced by fluctuations in atmospheric pressure. Gebert and Groengroft^11^ found that the amplitude of the gas pressure measured in two gas collection wells in an old German landfill exhibits a linear correlation with the amplitude of atmospheric pressure. Zhang et al.^12^ observed the gas pressure in the newly filled municipal solid waste (MSW) layer of the Wuxi landfill for more than 500 days, with results showing that the gas pressure varies with time, showing a single peak curve. The stratification of new and old waste layers is constantly occurring in operating landfills^12–14^. The variation of gas pressure over time in this landfill needs to be estimated by theoretical calculation.

At present, many scholars have obtained the gas pressure distribution in landfills through numerical^1,15–24^ and analytical^25–29^ calculations. Current research is mainly based on the theory to estimate the gas pressure in landfills as the data of gas pressure distribution in the field is rarely reported, and estimation of gas pressure through the combination of the field data and theoretical analysis is relatively scarce. Moreover, landfills in these studies are generally regarded as landfills with a homogeneously unsaturated waste layer^1,15–17,19,24–29^ or a continuously placed waste layer^20,22,23,28,29^. Findikakis and Leckie^15^, Findikakis et al.^16^, and Liu et al.^27^ considered the increasing stage of gas production rate, and Lu et al.^23^ considered the anisotropic ratio of gas permeability. The working conditions for the stratification of new and old waste and the stratification of saturated and unsaturated waste in an operating landfill were investigated by Zhang et al.^12^, but current research methods have not considered these two working conditions. If the gas pressure distribution in the landfill needs to be estimated, it is necessary to consider the increasing stage of gas production rate and the anisotropy ratio of gas permeability. In addition, the stratification of new and old waste and the stratification of saturated and unsaturated waste should also be considered.

Based on numerical methods, a one-dimensional gas transport model for estimating the gas pressure in a landfill with layered new and old waste and layered saturated and unsaturated waste is presented in this study. The factors considered in this model are as follow: firstly, the stratification of the length of time that waste has been degraded, gas permeability, and porosity; secondly, the anisotropy ratio of gas permeability; thirdly, the settlement caused by waste biodegradation; fourthly, the source term of gas production. The equation for quantifying settlement induced by waste biodegradation is presented, and the time of peak gas production rate is obtained by fitting the settlement of the new waste layer with this equation. The stratification of the unsaturated and saturated zones is considered by distinguishing the gas saturation in the different zones. The stratification of the new and old waste layers is considered by distinguishing the length of time that the waste has been degraded to produce gas in the different waste layers. The results of the numerical calculation are then compared with the gas pressure in the newly filled waste layer measured by Zhang et al.^12^. The validity of the calculation method and the theoretical model in simulating the gas pressure distribution in the newly filled waste layer is determined, and the evolution of the gas pressure distribution in the landfill with layered new and old waste layers is analyzed. The position of the maximum gas pressure and the significant sensitive parameter of the gas pressure distribution are found.

## Materials and methods

### Numerical model for gas transport

To establish a model for predicting gas pressure in landfills, it is assumed that gas transport in the MSW layer follows Darcy’s law, and settlement only occurs in the vertical direction. According to the mass conservation law, the net mass of gas flowing into and out of the unit body plus the mass of gas production equal to the variation of gas mass in the unit body, which can be represented by the following equation:

1 $$- \left( {\frac{{\partial \rho_{g} V_{x} }}{\partial x}dx + \frac{{\partial \rho_{g} V_{y} }}{\partial y}dy + \frac{{\partial \rho_{g} V_{z} }}{\partial z}dz} \right)dt + \rho_{g} Q_{G} dxdydzdt = \frac{{\partial \rho_{g} nS_{g} dxdydz}}{\partial t}dt$$

where *ρ*_*g*_ is the density of landfill gas (kg m^-3^); *V*_*x*_, *V*_*y*_ and *V*_*z*_ are the volumes of landfill gas entering the unit body along directions *ox*, *oy* and *oz* per unit time (m^3^ d^−1^), respectively; *Q*_*G*_ is the volume of gas produced by biodegradation per unit volume of waste per unit time (d^−1^); *n* is the porosity of waste; *S*_*g*_ is the gas saturation.

Following Darcy”s law, the following equation is obtained:

2 $$V_{i} = - \frac{{K_{gi} }}{{\mu_{g} }}P_{g,i} djdk$$

where *i*, *j* or k = *x*, *y*, or *z* (*i* ≠ *j* ≠ *k*); *K*_*gi*_ is the gas permeability in the direction *i* (*m*^*2*^); *μ*_*g*_ is the gas dynamic viscosity (Pa s); *P*_*g*_ is gas pressure (kPa).

When the settlement caused by waste biodegradation in landfills is considered, the following equation can be obtained:

3 $$\frac{\partial dz}{{\partial t}} = dz\frac{{d\varepsilon_{t} }}{dt}$$

where *ε*_*t*_ is the biodegradation settlement strain of the landfill.

According to the ideal gas law, the following equation can be obtained:

4 $$\rho_{g} = \frac{{\left( {P_{g} + P_{atm} } \right)M_{g} }}{RT}$$

where *P*_*atm*_ is atmospheric pressure (kPa); *M*_*g*_ is the molar mass of landfill gas (g mol^−1^); *R* is the gas constant (J (mol K)^−1^); *T* is waste temperature (℃).

According to the equation of waste biodegradation rate presented by Liu et al.^27^, and waste can be divided into easily degraded, moderately degraded, and difficultly degraded according to the biodegradability of the waste^19,30,31^. The volume of landfill gas generated by biodegradation per unit volume of waste per unit time can be presented, as is shown in the following equation:

5 $$Q_{G} = \rho L_{0} \sum\limits_{i = 1}^{3} {\omega_{i} \frac{{A_{Gi} }}{{B_{Gi} }}\left( {t + D_{Gi} } \right)e^{{ - \frac{{t + D_{Gi} }}{{B_{Gi} }}}} }$$

where *ρ* is the density of waste (kg m^-3^); *L*_0_ is the volume of landfill gas generated by biodegradable per unit mass of waste (m^3^ kg^−1^); *i* = 1, *i* = 2, and *i* = 3 represent easily degraded, moderately degraded and difficultly degraded component in waste, respectively; *ω*_*i*_ is the proportion of component; *A*_*Gi*_ is the parameter related to gas production rate (d^−1^); *B*_*Gi*_ is the time of peak gas production rate (d); *D*_*Gi*_ is the length of time that the waste has been degraded to produce gas (d).

**Figure 1:**
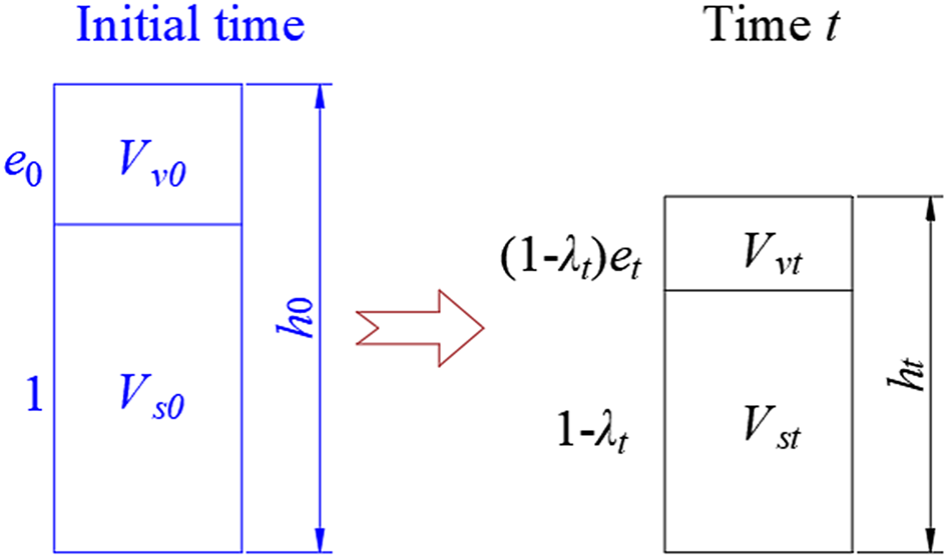
Schematic diagram of simplified landfill.

To facilitate the calculation of the settlement caused by waste biodegradation, the landfill is simplified into the schematic diagram shown in Fig. 1. Based on Fig. 1, the settlement strain of a landfill can be presented (Specific processes are shown in the Appendix), as is shown in the following equation:

6 $$\varepsilon_{t} = \sum\limits_{i = 1}^{3} {\left\{ {1 - \frac{1}{{e_{0} + 1}}\left[ {e_{0} - C_{c} \lg \left( {1 + \frac{t}{{t_{D} }}} \right) + 1} \right]\left[ {1 - A_{Gi} \times B_{Gi} \left( { - \frac{t}{{B_{Gi} }}e^{{ - \frac{t}{{B_{Gi} }}}} + 1 - e^{{ - \frac{t}{{B_{Gi} }}}} } \right)} \right]} \right\}}$$

where *λ*_*t*_ is waste biodegradation rate; *m*_*s*0_ is the waste mass at the initial moment; *m*_*st*_ is the waste mass at the moment *t*; *V*_*s*0_ is the waste volume at the initial moment; *V*_*st*_ is the waste volume at the moment *t*; *h*_0_ is the initial height of landfill; *h*_*t*_ is the height of landfill at time *t*; *e*_0_ is the initial void ratio; *e*_*t*_ is the void ratio at time *t*.

Substituting Eqs. (2)–(6) into Eq. (1), since the pressure of landfill gas is a very small value compared with the atmospheric pressure, then the pressure of landfill gas can be neglected when the sum of the gas pressure in landfills and the atmospheric pressure is calculating^29,30,32,33^. The gas pressure distribution in the landfill is a one-dimensional problem, because the landfill gas is only considered to migrate to the outside and into the old waste layer. Therefore, the following equation can be obtained:

7 $$\begin{gathered} \frac{{K_{gz} }}{{u_{g} }}\frac{{\partial^{2} P_{g} }}{{\partial z^{2} }} + \frac{1}{{u_{g} }}\frac{{\partial K_{gz} }}{\partial z}\frac{{\partial P_{g} }}{\partial z} + \rho L_{0} \sum\limits_{i = 1}^{3} {\omega_{i} \frac{{A_{Gi} }}{{B_{Gi} }}\left( {t + D_{Gi} } \right)e^{{ - \frac{{t + D_{Gi} }}{{B_{Gi} }}}} } - \hfill \\ nS_{g} \left\{ \begin{gathered} 1 + \frac{1}{{e_{0} + 1}}\left[ {e_{0} - C_{c} \lg \left( {1 + \frac{t}{{t_{D} }}} \right) + 1} \right]\frac{{A_{Gi} t}}{{B_{Gi} }}e^{{ - \frac{t}{{B_{Gi} }}}} \hfill \\ + \frac{{C_{c} }}{{\left( {e_{0} + 1} \right)\left( {t + t_{D} } \right)\ln 10}}\left[ {1 - A_{Gi} \times B_{Gi} \left( { - \frac{t}{{B_{Gi} }}e^{{ - \frac{t}{{B_{Gi} }}}} + 1 - e^{{ - \frac{t}{{B_{Gi} }}}} } \right)} \right] \hfill \\ \end{gathered} \right\}{ = }\frac{{nS_{g} }}{{P_{atm} }}\frac{{\partial P_{g} }}{\partial t} \hfill \\ \end{gathered}$$

Equation (7) is the one-dimensional transient basic difference equation of gas transport in the MSW layer.

The following equation can be obtained by differencing Eq. (7):

8 $$f_{G1} \frac{{P_{gk - 1}^{t} - 2P_{gk}^{t} + P_{gk + 1}^{t} }}{{h_{z}^{2} }} + f_{G2} \frac{{P_{gk + 1}^{t} - P_{gk - 1}^{t} }}{{2h_{z} }} + f_{G3} = f_{G4} \frac{{P_{gk}^{t} - P_{gk}^{t - 1} }}{\tau }$$

where $f_{G1} = \frac{{K_{gz} }}{{\mu_{g} }}$; $f_{G2} = \frac{1}{{\mu_{g} }}\frac{{\partial K_{gz} }}{\partial z}$; $$\begin{gathered} f_{G3} = \rho L_{0} \sum\limits_{i = 1}^{3} {\omega_{i} \frac{{A_{Gi} }}{{B_{Gi} }}\left( {t + D_{Gi} } \right)e^{{ - \frac{{t + D_{Gi} }}{{B_{Gi} }}}} } - \hfill \\ nS_{g} \left\{ \begin{gathered} 1 + \frac{1}{{e_{0} + 1}}\left[ {e_{0} - C_{c} \lg \left( {1 + \frac{t}{{t_{D} }}} \right) + 1} \right]\frac{{A_{Gi} t}}{{B_{Gi} }}e^{{ - \frac{t}{{B_{Gi} }}}} \hfill \\ +\frac{{C_{c} }}{{\left( {e_{0} + 1} \right)\left( {t + t_{D} } \right)\ln 10}}\left[ {1 - A_{Gi} \times B_{Gi} \left( { - \frac{t}{{B_{Gi} }}e^{{ - \frac{t}{{B_{Gi} }}}} + 1 - e^{{ - \frac{t}{{B_{Gi} }}}} } \right)} \right] \hfill \\ \end{gathered} \right\} \hfill \\ \end{gathered}$$; $f_{G4} = \frac{{nS_{g} }}{{P_{atm} }}$;*h*_*z*_ is the step length in the vertical direction; *τ* is the step length of time.

Equation (8) is the difference equation of a one-dimensional transient difference scheme for gas transport in a landfill. The difference equation is in the classical implicit format, indicating that the difference equation can be solved via iterative methods.

### Verification of numerical method

In this study, the gas pressure in the new and old waste layers is calculated based on the defined numerical calculation method. In order to verify the reliability of this method, the mathematical model of gas transport presented by Li et al.^29^ is calculated with new method, and the numerical results are compared with the original analytical results. The mathematical model of gas transport presented by Li et al.^29^ is shown in Eq. (9), respectively:

9 $$\left\{ \begin{gathered} {\text{Governing}}\;{\text{equation:}}\;\frac{{\partial P_{g} }}{\partial t} = \alpha_{L} \frac{{\partial^{2} P_{g} }}{{\partial z^{2} }} + \beta_{L} e^{{ - k_{L} \left( {t + {\raise0.7ex\hbox{$z$} \!\mathord{\left/ {\vphantom {z {r_{L} }}}\right.\kern-\nulldelimiterspace} \!\lower0.7ex\hbox{${r_{L} }$}}} \right)}} \hfill \\ {\text{Initial}}\;{\text{condition: }}P_{g} (z,t = 0) = 0 \hfill \\ {\text{Upper}}\;{\text{boundary}}:\;P_{g} (z = 0,t) = 0 \hfill \\ {\text{Lower}}\;{\text{boundary}}:\;\frac{{\partial P_{g} (z = H_{L} ,t)}}{\partial z} = 0 \hfill \\ \end{gathered} \right.$$

where $\alpha_{L} { = }\frac{{P_{atm} K_{G} }}{{n_{gL} \mu_{L} }}$; $\beta_{L} = \frac{{RT_{L} }}{{n_{{g_{L} }} \omega_{L} }}L_{g} k_{L}$; *P*_*g*_ is gas pressure (kPa); *P*_*atm*_ is atmospheric pressure (101.3 kPa); *K*_*G*_ is gas permeability (6.9 × 10^–13^ m^2^); *n*_*g*_ is gas content (0.5); *μ*_*L*_ is gas dynamic viscosity (1.37 × 10^–5^ kg (m s) ^−1^); *R* is gas constant (8.31 J (K mol) ^−1^); *T*_*L*_ is gas temperature (298 ℃); *ω*_*L*_ is the molar mass of gas (0.03 g mol^−1^); *L*_*g*_ is the mass of gas produced by the waste of unit volume (230 kg m^-3^); *k*_*L*_ is the constant of gas production rate (0.1 year^−1^); *r*_*L*_ is the waste placing rate (3 m year^−1^); *H*_*L*_ is the thickness of landfill (30 m).

When Eq. (9) is differenced, the numerical model for calculating the gas pressure in the study of Li et al.^29^ can be obtained as follow:

10 $$\left\{ \begin{gathered} {\text{Governing}}\;{\text{equation:}}\;\frac{{P_{gk}^{t} - P_{gk}^{t - 1} }}{\tau } = \alpha_{L} \frac{{P_{g}^{t} - 2P_{gk}^{t} + P_{gk + 1}^{t} }}{{h_{z}^{2} }} + \beta_{L} e^{{ - k_{L} \left[ {t + {\raise0.7ex\hbox{${h_{z} \left( {k - 1} \right)}$} \!\mathord{\left/ {\vphantom {{h_{z} \left( {k - 1} \right)} {r_{L} }}}\right.\kern-\nulldelimiterspace} \!\lower0.7ex\hbox{${r_{L} }$}}} \right]}} \hfill \\ {\text{Initial}}\;{\text{condition: }}P_{gk}^{t = ot\left( 0 \right)} = 0 \hfill \\ {\text{Upper}}\;{\text{boundary}}:\;P_{gk = oz\left( 0 \right)}^{t} = 0 \hfill \\ {\text{Lower}}\;{\text{boundary}}:\;P_{{gk = oz\left( {H_{L} } \right)}}^{t} = P_{{gk - 1 = oz\left( {H_{L} } \right) - 1}}^{t} \hfill \\ \end{gathered} \right.$$

where, *h*_*z*_ is the step length in the vertical direction; *τ* is the step length of time; *oz*(*z*) and *ot*(*t*) are the node functions in the vertical direction and time, respectively.

**Figure 2:**
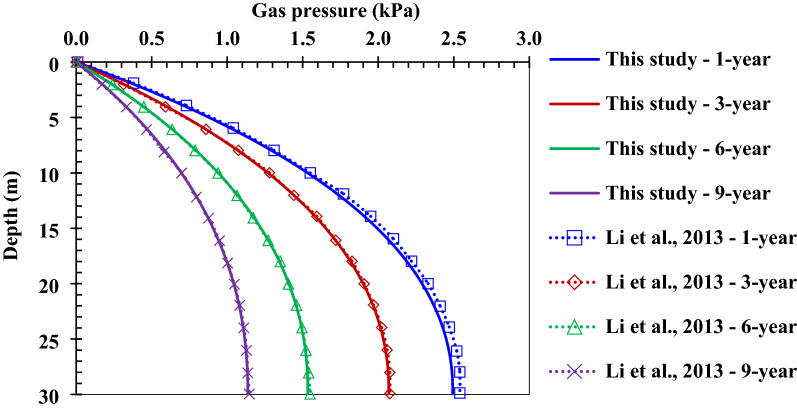
Comparison of the calculation values of gas pressure in this study and the value calculated by Li et al.^29^.

The gas pressure distribution in the landfill described by Li et al.^29^ is obtained by the numerical calculation method, as is shown in Fig. 2. It can be found that the curve of gas pressure with depth obtained by using the numerical calculation method in this study closely matches the curve of gas pressure with depth obtained by Li et al.^29^ using their analytical method, illustrating the reliability of the numerical method used in this study.

## Results and discussion

### Gas pressure distribution in the Wuxi landfill

Wuxi landfill, where Zhang et al.^12^ conducted their field test, is located in the city of Wuxi, China, approximately 100 km northwest of Shanghai. The average annual rainfall is approximately 1900 mm, and the atmospheric temperature ranges from − 9 to 39°C^12^. Wuxi landfill is a typical valley-type landfill, constructed in 1995. Designed to accommodate 4.20 million m^3^ of MSW. During the year 2016, 2200 t d^−1^ of MSW were placed in this landfill. The organic waste mainly includes food, textile, paper and wood, their total content is approximately 48.9%^12^. Zhang et al.^12^ monitored the gas pressure in the new waste layer from January 18, 2016 to June 18, 2017. The gas pressure is obtained by measuring the pressure at the head of the monitoring well using a micromanometer.

**Figure 3:**
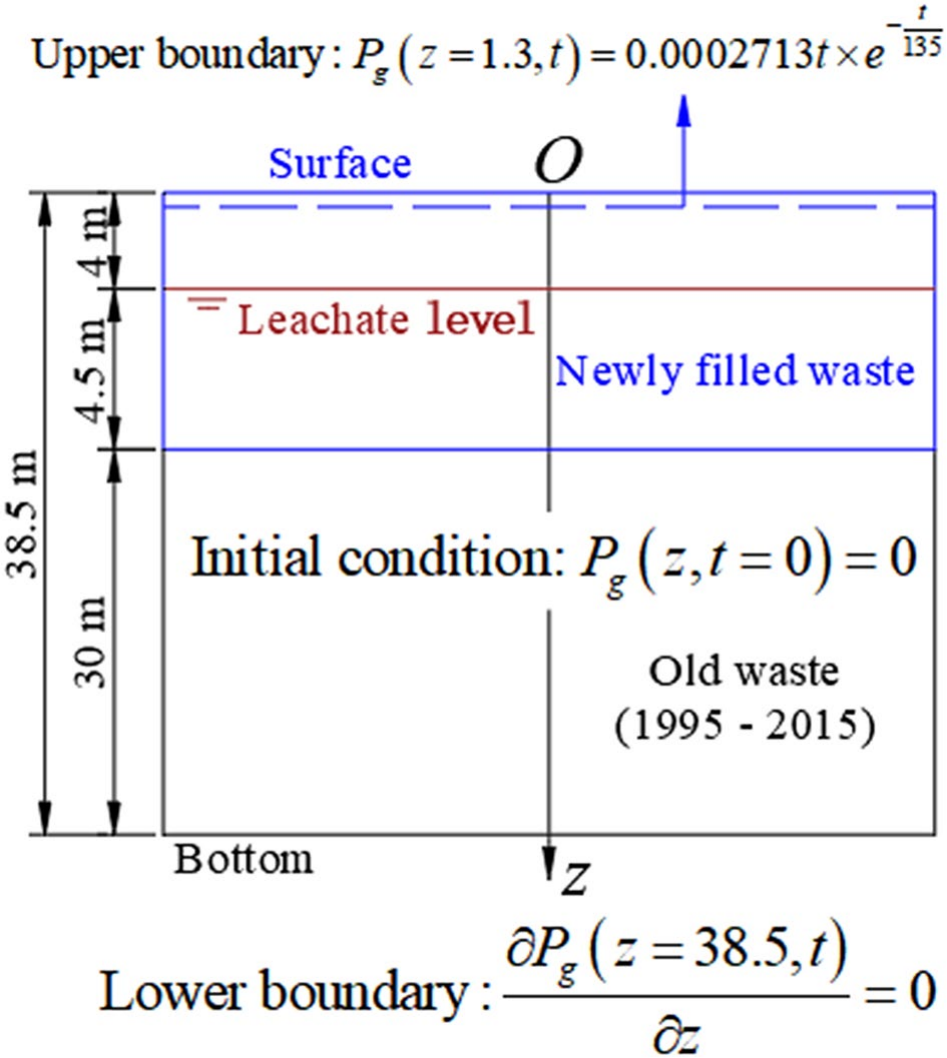
Physical model for gas transport in the Wuxi landfill.

The leachate drainage and liner systems were built at the bottom of the Wuxi landfill. However, there is not a landfill gas extraction system at the bottom. Therefore, the boundary condition at the bottom of the landfill can be considered as an impermeable boundary condition (Neumann boundary condition) in the numerical calculation. Generally, when the gas pressure in an uncovered landfill is calculated, the upper boundary is regarded as the atmospheric pressure, that is, the relative gas pressure is zero^16,17,19,20,22,24,26,28,29^. However, in order to reasonably simulate the upper boundary of the Wuxi landfill, the gas pressure at the shallowest depth (depth: 1.3 m) observed by Zhang et al.^12^ is used as the upper boundary (Dirichlet boundary condition). The gas pressure at this depth is fitted with a single peak function, and the fitting result is shown in the top of Fig. 3. The initial gas pressure is 0 kPa, because the method for burying gas collection and monitoring wells during a field test is to excavate the waste body^12^. The physical model for simulating gas transport in the Wuxi landfill is shown in Fig. 3.

According to Eq. (7) and Fig. 3, the following mathematical model can be obtained:

11 $$\left\{ \begin{gathered} {\text{Governing}}\;{\text{equation:}}\;\frac{{K_{gz} }}{{u_{g} }}\frac{{\partial^{2} P_{g} }}{{\partial z^{2} }} + \frac{1}{{u_{g} }}\frac{{\partial K_{gz} }}{\partial z}\frac{{\partial P_{g} }}{\partial z} + \rho L_{0} \sum\limits_{i = 1}^{3} {\omega_{i} \frac{{A_{Gi} }}{{B_{Gi} }}\left( {t + D_{Gi} } \right)e^{{ - \frac{{t + D_{Gi} }}{{B_{Gi} }}}} } \hfill \\ - nS_{g} \left\{ \begin{gathered} 1 + \frac{1}{{e_{0} + 1}}\left[ {e_{0} - C_{c} \lg \left( {1 + \frac{t}{{t_{D} }}} \right) + 1} \right]\frac{{A_{Gi} t}}{{B_{Gi} }}e^{{ - \frac{t}{{B_{Gi} }}}} \hfill \\ +\frac{{C_{c} }}{{\left( {e_{0} + 1} \right)\left( {t + t_{D} } \right)\ln 10}}\left[ {1 - A_{Gi} \times B_{Gi} \left( { - \frac{t}{{B_{Gi} }}e^{{ - \frac{t}{{B_{Gi} }}}} + 1 - e^{{ - \frac{t}{{B_{Gi} }}}} } \right)} \right] \hfill \\ \end{gathered} \right\}{ = }\frac{{nS_{g} }}{{P_{atm} }}\frac{{\partial P_{g} }}{\partial t} \hfill \\ \hfill \\ {\text{Initial}}\;{\text{condition: }}P_{g} \left( {z,t = 0} \right) = 0 \hfill \\ {\text{Upper}}\;{\text{boundary}}:\;P_{g} \left( {z = 1.3,t} \right) = 0.0002713t \times e^{{ - \frac{t}{135}}} \hfill \\ {\text{Lower}}\;{\text{boundary}}:\;\frac{{\partial P_{g} \left( {z = 38.5,t} \right)}}{\partial z} = 0 \hfill \\ \end{gathered} \right.$$

When Eq. (11) is differenced, and according to Eq. (8), the following mathematical model of difference scheme can be obtained:

12 $$\left\{ \begin{gathered} {\text{Governing}}\;{\text{equation:}}\;f_{G1} \frac{{P_{gk - 1}^{t} - 2P_{gk}^{t} + P_{gk + 1}^{t} }}{{h_{z}^{2} }} + f_{G2} \frac{{P_{gk + 1}^{t} - P_{gk - 1}^{t} }}{{2h_{z} }} + f_{G3} = f_{G4} \frac{{P_{gk}^{t} - P_{gk}^{t - 1} }}{\tau } \hfill \\ {\text{Initial}}\;{\text{condition: }}P_{gk}^{t = ot(0)} = 0 \hfill \\ {\text{Upper}}\;{\text{boundary}}:\;P_{gk = oz(1.3)}^{t} = 0.0002713\left[ {ot(t) - 1} \right]\tau \times e^{{ - \frac{{\left[ {ot(t) - 1} \right]\tau }}{135}}} \hfill \\ {\text{Lower}}\;{\text{boundary}}:\;P_{gk = oz(38.5)}^{t} = P_{gk = oz(38.5) - 1}^{t} \hfill \\ \end{gathered} \right.$$

where *τ* is the step length of time; *ot*(*t*) and *oz*(*z*) are the node functions in time and the vertical direction, respectively.

The gas pressure distribution in the monitoring test conducted by Zhang et al.^12^ is studied, and the layout of the field test site can be viewed in the paper of Zhang et al.^12^. In order to select reasonable calculation parameters, the parameters which are suitable for the Wuxi landfill are selected. The parameters used in the numerical calculation are shown in Table 1. In the process of numerical calculation, the step length in the vertical direction is 0.5 m and the step length of time is one day. The equation of gas transport in this study is a parabolic equation, and its difference scheme (Eq. 8) is a classical implicit scheme. Therefore, the iterative method is used to solve the difference equation with absolute convergence using any mesh ratio. The stratification of the unsaturated and saturated waste is realized by distinguishing the gas saturation above and below the leachate level. The stratification of the new and old waste is realized by distinguishing the length of time that waste has been degraded to produce gas in the new and old waste layers. In addition, in order to simulate the actual landfill, the variations of gas permeability and porosity with depth, the anisotropic ratio of gas permeability, and the settlement caused by waste biodegradation were also considered.

**Table 1:** Parameters of gas transport model in the Wuxi landfill.

Parameter	Value	Value from reference	References
*ρ* (kg m^−3^)	700	700	^12^
*n*	$- 0.0042z + 0.7309$	$- 0.0042z + 0.7309$^a^	^34^
*S*_*g*_	$$\begin{aligned} S_{g} & = 1 - \frac{{\omega_{m} \rho }}{{n\rho_{w} }} \\ & = \left\{ {\begin{array}{*{20}c} {1 - \frac{0.349 \times 700}{{n \times 1000}} = 1 - 0.2443 \times \frac{1}{n}, {\text{Unsaturated}}\;{\text{zone}}} \\ {1 - \frac{0.591 \times 700}{{n \times 1000}} = 1 - 0.4137 \times \frac{1}{n}, {\text{Saturated}}\;{\text{zone}} } \\ \end{array} } \right. \\ \end{aligned}$$^b^	–	–
*D*_*G*_ (d)	$$D_{G} = \left\{ {\begin{array}{*{20}l} {0,} \hfill & { {\text{New}}\;{\text{waste}}\;{\text{layer}}} \hfill \\ {40 + \frac{z - 8.5}{{1.5}} \times 365,} \hfill & { {\text{Old}}\;{\text{waste}}\;{\text{layer}}} \hfill \\ \end{array} } \right.$$^c^	–	–
*K*_*gx*_ (m^2^)	$$\begin{gathered} 0.01 \times 8 \times 10^{ - 8} n^{19.513} \hfill \\ + 0.99 \times 6 \times 10^{ - 4} n^{56.306} \hfill \\ \end{gathered}$$^d^	$$\left\{ \begin{gathered} 8 \times 10^{ - 8} n^{19.513} {,}\;{\text{Upper}}\;{\text{limit}} \hfill \\ 6 \times 10^{ - 4} n^{56.306} {,}\;{\text{Lower}}\;{\text{limit}} \hfill \\ \end{gathered} \right.$$^e^	^35–38^
*K*_*gx*_*/K*_*gz*_	3	3	^30,32,33^
*μ*_*g*_ (Pa s)	1.45 × 10^–5^	1.36 × 10^–5^ − 1.83 × 10^–5^	^33,39,40^
*L*_*0*_ (m^3^ kg^−1^)	0.112	0.112f.	^41^
*P*_*atm*_ (kPa)	101.325	–	–
*ω*_1_, *ω*_2_ and *ω*_3_	0.15, 0.55 and 0.30	0.15, 0.55 and 0.30	^19,30,31,42^
*A*_*G*1_, *A*_*G*2_ and *A*_*G*3_ (d^−1^)	1.03 × 10^–3^, 1.71 × 10^–4^, 1.28 × 10^–4^	1.03 × 10^–3^, 1.71 × 10^–4^, 1.28 × 10^–4^	
*B*_*G*1_, *B*_*G*2_ and *B*_*G*3_ (d)	80, 160 and 350^g^	–	–
*t*_*D*_ (d)	30	30	^43,44^
*C*_*c*_	0.065	0.03–0.1	^43,44^

^a^*n* is obtained by fitting the porosity in the Suzhou landfill (about 30 km from the Wuxi landfill), where *z* is depth;

^b^Where *ω*_*m*_ is the mass moisture content of waste (the average mass moisture content in saturated and unsaturated areas are 59.1% and 34.9%^45^, respectively); *ρ*_*w*_ is the water density;

^c^In order to simulate the gas pressure in the monitoring well, which is placed after the waste body is excavated, the length of time that waste in the new waste layer has been degraded to produce gas is 0; 40 is the average length of time for the existence of new waste layer (d); 8.5 is the thickness of the new waste layer (m); 1.5 is the average height for placing the waste in the old waste layer each year (m);

^d^The fitting curve through the gas permeability in the Suzhou landfill^35^ is selected as the curve of gas permeability in the Wuxi landfill;

^e^A power function was used to fit the relationship between gas permeability and porosity measured by Stoltz et al.^36^ to obtain the upper and lower limits of gas permeability in landfills;

^f^According to the statistic on the gas production potential of waste in different landfills^41^, the gas production potential of waste in the Hangzhou landfill (about 140 km from the Wuxi landfill) is found;

^g^The settlement strain obtained from Eq. (6) is multiplied by the thickness of the new waste layer (8.59 m) to fit the settlement of the new waste layer in the test area, as is shown in Fig. 4.

The variation of gas pressure with time in the newly filled MSW layer of the Wuxi landfill was obtained by numerical calculation, as is shown in Fig. 5. Both the theoretical calculation value and the monitoring value of gas pressure show the single peak trend, which is similar to the trend of waste biodegradation rate^27^. It is worth noting that the gas pressure in #0 well (extraction well) is the mixed gas pressure within the entire perforated pipe (length: 7.7 m). In order to calculate the gas pressure in the gas collection well, the gas pressure in the waste body is integrated into the direction of the length of the perforated pipe. Then the result obtained by integration is divided by the length of the perforated pipe, and the average gas pressure in the gas collection well is obtained, as is shown in Fig. 5a. When the leachate level is always higher than the bottom of the monitoring well (e.g. #6 well), or when the bottom of the monitoring well (e.g. #3well and #5 well) is submerged due to the increase of leachate level in the later period, the gas pressure below the leachate level is not monitored in the field test of Zhang et al.^12^. However, the gas pressure below the leachate level in the landfill can be theoretically calculated, as is shown in Fig. 5b–d. The calculation value is compared with the field test value in Fig. 5, it can be also found that the variation of gas pressure in the newly filled MSW layer with time is better simulated by the calculation method and numerical model presented in this study.

**Figure 4:**
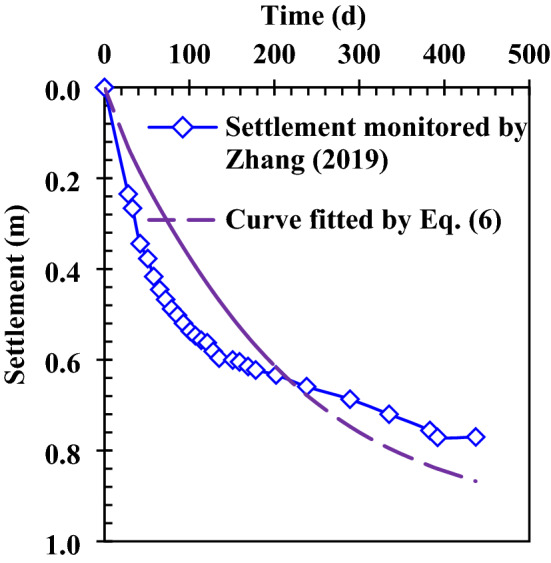
Settlement of newly filled MSW layer varies with time.

The variation of gas pressure with height in the Wuxi landfill is shown in Fig. 6. The rate of gas production is faster in areas with new waste biodegradation, which leads to an increase in gas pressure within the newly filled MSW layer that increases with depth. As porosity^34,46^ and gas permeability^35^ decrease with depth in landfills, the pore volume and migratory ability of the gas, respectively, also decrease with depth, which ultimately increases gas pressure with depth. According to the ideal gas law, the gas pressure increases with depth under the assumption that the gas production yield of new waste is constant.

**Figure 5:**
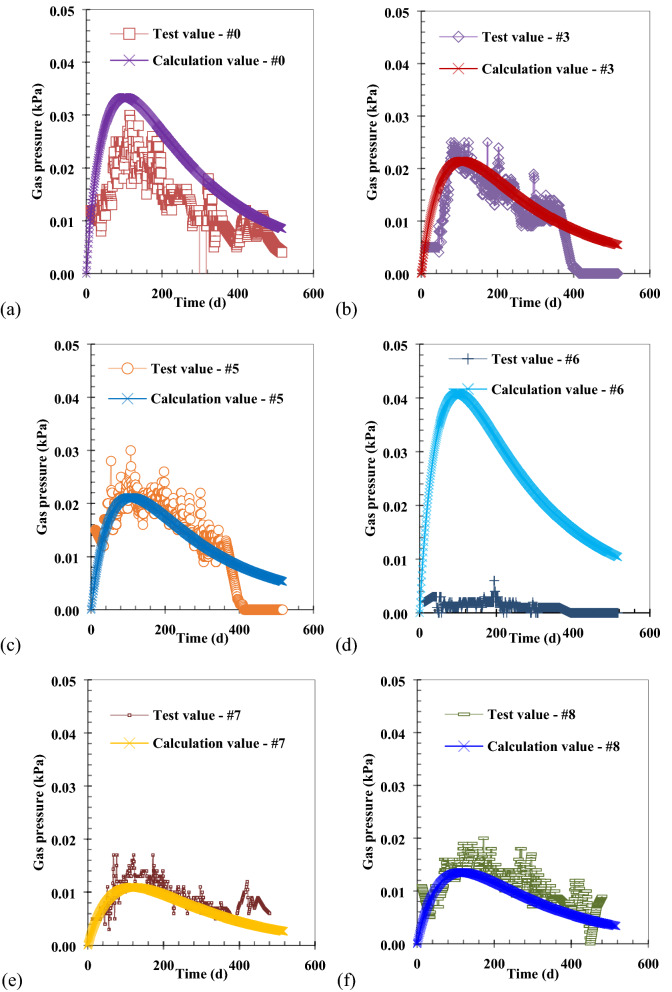
Comparison of calculation and test values of gas pressure with time at the bottom of different wells: (**a**) #0 well (Depth: 8.74 m); (**b**) #3 well (Depth: 3.64 m); (**c**) #5 well (Depth: 3.58 m); (**d**) #6 well (Depth: 6.56 m); (**e**) #7 well (Depth: 1.34 m); (**f**) #8 well (Depth: 2.05 m).

The waste in the old MSW layer has been buried for a longer period time, and the gas production rate decreases with depth. The gas pressure in the old MSW layer decreases with depth in the earlier period. Therefore, it can be inferred that the maximum gas pressure at this stage occurs where the waste biodegradation rate is not high. According to the result of calculation, this position is in the old MSW layer close to the boundary of the new and old waste layers. At the same time, as the landfill gas in the newly filled MSW layer migrates to the old MSW layer, the gas pressure in the old waste layer begins to increase.

**Figure 6:**
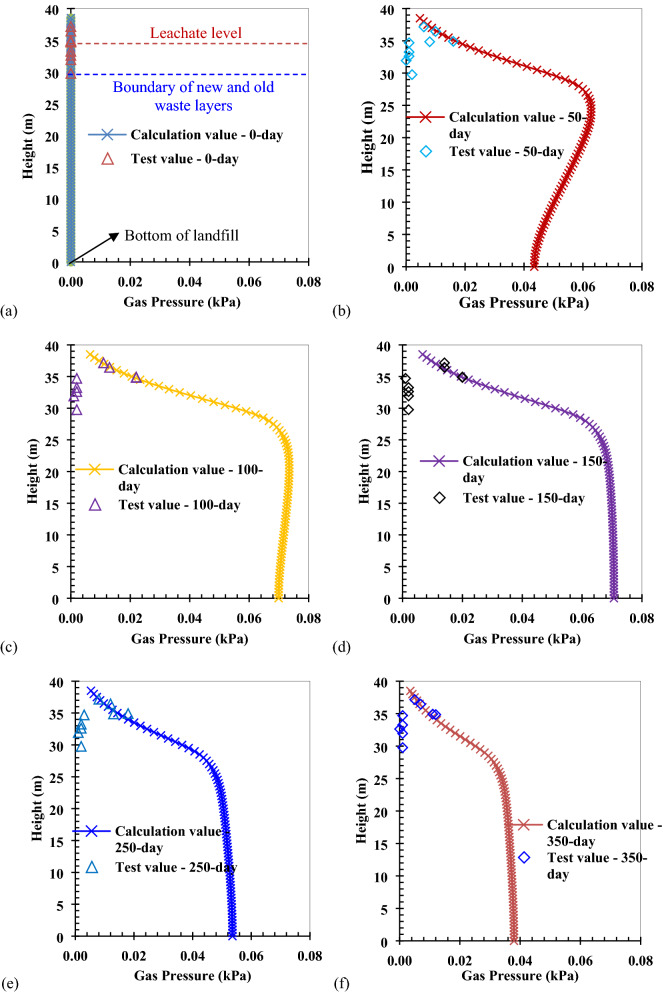
Comparison of calculation and test values of gas pressure with height on the different time: (**a**) day 0; (**b**) day 50; (**c**) day 100; (**d**) day 150; (**e**) day 250; (**f**) day 350.

As the waste biodegradation rate in the newly filled waste later begins to decrease, the rate of gas pressure increase begins to slow until it ultimately reaches its peak. Due to the migration of landfill gas from the position of high gas pressure to the old MSW layer with low gas pressure, the gas pressure in this layer begins to increase with depth. Therefore, the maximum gas pressure in the later period appears at the bottom of landfill. However, the maximum gas pressure in this period is less than that in the earlier period. Because the landfill gas slowly migrates from the position of higher pressure to the position of lower pressure in the later period, resulting in the gas pressure in the landfill to slowly decrease. The gas pressure gradient in the old waste layer is less than that in the new waste layer, because the waste biodegradation rate in the old waste layer is lower than that in the new waste layer. The gas pressure below the leachate level was not monitored in the field test conducted by Zhang et al.^12^. However, the gas pressure below the leachate level can be obtained by theoretical calculation. It can be also found from Fig. 6 that the calculation method and the numerical model used in this study better reflect the gas pressure distribution in the new and old waste layers by comparing the calculation value with the test value.

### Sensitivity analysis

Applying the upper and lower limits of gas permeability relative to porosity, and the equation relating porosity to depth in Table 1, the upper and lower limits of gas permeability varying with depth can be obtained, as is shown in Eq. (13):

13 $$K_{gx} = \left\{ \begin{gathered} 8 \times 10^{ - 8} \times \left( { - 0.0042z + 0.7309} \right)^{19.513} \left( {m^{2} } \right),\;{\text{Upper limit}} \hfill \\ 6 \times 10^{ - 4} \times \left( { - 0.0042z + 0.7309} \right)^{56.306} \left( {m^{2} } \right),\;{\text{Lower limit}} \hfill \\ \end{gathered} \right.$$

The comparison of gas pressure distribution under the conditions of different gas permeabilities is shown in Fig. 6a. It can be found that the smaller the gas permeability, the greater the gas pressure. Because if the gas permeability is smaller, the velocities of gas transport in the horizontal and vertical directions are also smaller. Thus, it becomes difficult for landfill gas to migrate, resulting in larger gas pressure at each subsequent depth. When the gas pressure distribution in a landfill is calculated, the gas permeability that is suitable for the estimated landfill should be selected, and the variation of gas permeability with depth should be considered. If the selected gas permeability is lower than the actual one, it will cause the estimated gas pressure to be larger.

**Figure 7:**
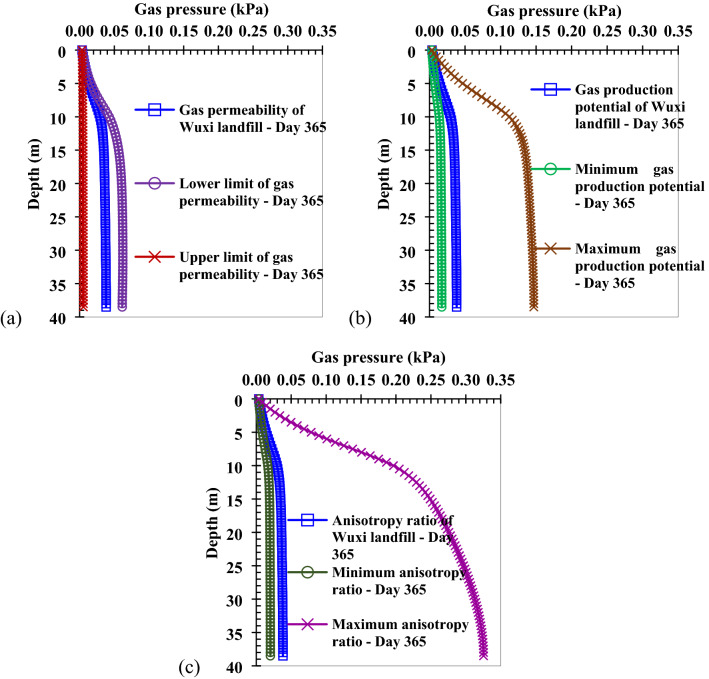
Comparison of gas pressure distribution under the conditions of different sensitive parameters: (**a**) gas permeability; (**b**) gas production potential (43.3–469 L kg^−1^); (**c**) anisotropy ratio (1.5–20).

The gas production potential of waste is closely related to gas production, Gao et al.^41^ and Zhan et al.^47^ summarized the range of gas production potential, and found this to range from 43.3 to 469 L kg^−1^. The comparison of the gas pressure distribution under the conditions of different gas production potentials is shown in Fig. 7b. It can be found that the greater the gas production potential, the greater the gas pressure in the landfill. Because if the gas production potential is larger, the concentration of landfill gas produced by waste biodegradation per unit mass is also larger, thus, the gas pressure of landfill gas increases. In the control equation of gas transport (Eq. (7)), larger gas production potential makes the source term of gas production greater, which causes a larger value of gas pressure to be quantified. When the gas pressure distribution in a landfill is calculated, it is necessary to select a gas production potential that relates to actual landfill being studied. If the selected gas production potential is greater than the actual one, it will cause the estimated gas pressure to be overestimated.

The anisotropy ratio of gas permeability (gas permeability in the horizontal direction to gas permeability in the vertical direction) has a great influence on gas pressure^32^. The range of the reported anisotropy ratio of gas permeability is between 1.5 and 20^32,39,48,49^. The comparison of the gas pressure distribution under the conditions of different anisotropy ratios is shown in Fig. 7c. It can be found that the greater the anisotropy ratio, the greater the gas pressure in the landfill. Because if the anisotropy ratio is larger, the vertical gas permeability is relatively small, making the vertical velocity of gas transport is small as well. This, in turn, makes the resultant velocity of gas transport relatively small, and the landfill gas is more difficult to migrate, ultimately resulting in greater gas pressure with depth. When the gas pressure distribution in a landfill is calculated, it is necessary to select the anisotropy ratio of gas permeability that is suitable for the estimated landfill. If the selected anisotropy ratio is greater than the actual one, it will cause the estimated gas pressure to be overestimated.

Using the mathematical model of gas transport developed in this study, the main sensitive parameters to the quantification of gas pressure were analyzed. A larger gas production potential, larger anisotropy ratio, and smaller gas permeability make the gas pressure in a landfill larger, which is beneficial for the collection of landfill gas. However, greater gas pressures adversely affect the stability of landfills and increase the possibility of landfill gas emissions. When gas permeability, gas production potential, and anisotropy ratio increase by 1%, the maximum gas pressures increase by − 0.02%, 0.76%, and 1.22%, respectively. Therefore, the gas pressure is most sensitive to the anisotropy ratio of gas permeability. It is very important to select the parameters that are suitable for the specific landfill when the gas pressure distribution is estimated. In particular, the irrationality of the anisotropy ratio will cause a large error in the estimation of gas pressure.

## Conclusions

A one-dimensional gas transport model was established for gas response in a landfill with layered new and old waste. The model takes into account the variation of gas permeability with depth, the anisotropy ratio of gas permeability, and the settlement caused by waste biodegradation. Besides, a single peak model for gas generation is used as the source term of gas production in this model. The stratification of the unsaturated and saturated zones is taken into account by distinguishing the difference in gas saturation. The layering of the new and old waste layers is considered by distinguishing the different length of time that waste has been degraded to produce gas. The time of peak gas production rate is obtained by fitting the settlement of the newly filled MSW layer with the biodegradation settlement equation presented in this study.

Based on the numerical calculation method, the gas pressure distribution in the new and old waste layers is obtained. After the results of calculation are compared with the test result, the following conclusions are found: (1) the gas pressure distribution in the landfill with layered new and old waste is better simulated using the gas transport model and the calculation method developed in this study; (2) the time of peak gas production rate fitted by biodegradation settlement is relatively reliable; (3) the maximum gas pressure occurs in the old waste layer near the boundary between new and old waste layers in the earlier period, but moves to the bottom of landfill in the later period. (4) The anisotropy ratio is a more sensitive parameter influencing the gas pressure distribution. In this study, the gas transport model, the calculation method, and the selection method of parameters provide a theoretical basis for evaluating the variation of gas pressure in a landfill with layered new and old waste.

## Supplementary Information

Supplementary Information
