# Supplementary material for: Simulation of gas transport in a landfill with layered new and old municipal solid waste
Source: Sci Rep. 2021 May 3;11:9436. doi: 10.1038/s41598-021-88858-5 (PMC8093249; doi:10.1038/s41598-021-88858-5)
Supplement: Supplementary file 1 — Supplementary Information [file 41598_2021_88858_MOESM1_ESM.docx]

## Appendix

The derivation process of Eq. (6) is described as follows.

The expression form of waste biodegradation rate is shown in the following equation:

$\lambda_{t}=\frac{m_{s0}-m_{st}}{m_{s0}}=\frac{\rho V_{s0}-\rho V_{st}}{\rho V_{s0}}=\frac{V_{s0}-V_{st}}{V_{s0}}$ (14)

The following equation can be obtained from Eq. (14):

$V_{st}=\left(1-\lambda_{t}\right)V_{s0}$ (15)

According to Eq. (15), a schematic diagram of a simplified landfill at time *t* can be obtained, as is shown in Fig. 1.

Because it is assumed that settlement only occurs in the vertical direction, the following equation can be obtained:

$\frac{h_{0}}{e_{0}+1}=\frac{h_{t}}{\left(e_{t}+1\right)\left(1-\lambda_{t}\right)}$ (16)

Thus, the settlement strain of landfill is shown in the following equation:

$\varepsilon_{t}=\frac{h_{0}-h_{t}}{h_{0}}=1-\frac{\left(e_{t}+1\right)\left(1-\lambda_{t}\right)}{e_{0}+1}$ (17)

According to the second compression model presented by Sowers^43^, the variation of void ratio can be obtained as follows:

$\Delta e=e_{0}-e_{t}=C_{c}\lg\left(1+\frac{t}{t_{D}}\right)$ (18)

where Δ*e* is the variable quantity of void ratio; *C_c_* is secondary compressibility; *t* is time; *t_D_* is the length of time in which the main compression is completed.

Substituting Eq. (18) into Eq. (17), and according to the equation of waste biodegradation rate presented by Liu et al.^27^, the settlement strain of landfill can be obtained, as is shown in Eq. (6).
